# The rapid detection of cefotaxime-resistant *Enterobacteriaceae* by HPLC

**DOI:** 10.4155/fsoa-2016-0042

**Published:** 2016-09-09

**Authors:** Andrew M Robinson, Natalie J Medlicott, James E Ussher

**Affiliations:** 1 Department of Microbiology & Immunology, University of Otago, Dunedin, New Zealand; 2 School of Pharmacy, University of Otago, Dunedin, New Zealand; 3 Southern Community Laboratories, Dunedin, New Zealand

**Keywords:** AmpC β-lactamases, cephalosporin resistance, *Enterobacteriaceae*, extended-spectrum β-lactamases (ESBL), HPLC

## Abstract

**Aim:**

Antibiotic resistance mediated by extended-spectrum β-lactamases (ESBL) and AmpC β-lactamases is widespread and increasingly common, often rendering empiric antibiotic therapy ineffective. In septicemia, delays in initiating effective antibiotic therapy are associated with worse clinical outcomes. With current phenotypic antimicrobial susceptibility testing methods, there is often a delay of 18–24 h before the susceptibility of an isolate is known.

**Results:**

Using an HPLC assay, breakdown of the third-generation cephalosporin cefotaxime by ESBL- and AmpC- β-lactamase-producing organisms could be detected within 90 min with 86.4% sensitivity and 100% specificity; sensitivity for ESBL detection was 100%.

**Conclusion:**

This assay could be readily established in any clinical laboratory with an HPLC to rapidly detect ESBL-producing *Enterobacteriaceae*.

## Lay abstract

In bloodstream infections, early initiation of effective antibiotics is critical. However, with increasing antimicrobial resistance empirical therapy may not be effective. Therefore rapid identification of resistant bacteria is required. Here we describe an assay that can detect resistant gram-negative bacteria within 90 min. Enteric gram-negative bacteria, including *Escherichia coli*, resistant to the extended-spectrum cephalosporin cefotaxime, could rapidly be identified by using HPLC to detect the breakdown of cefotaxime. This assay could reduce the time to detect resistant bacterial strains by almost a day.

**Figure 1.**
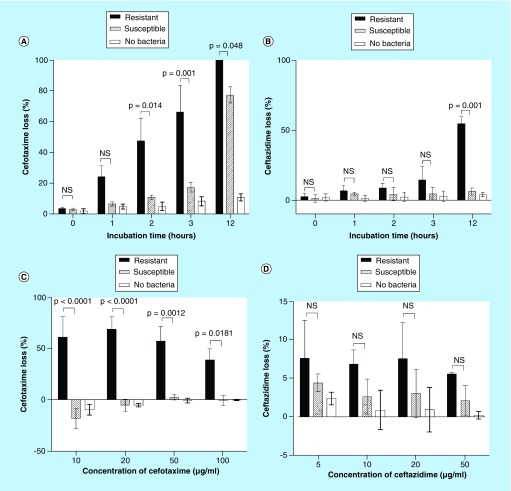
**Detection of extended-spectrum β-lactamases-mediated hydrolysis of cefotaxime and ceftazidime by HPLC.** **(A & B)** Optimization of incubation time for detection of cefotaxime and ceftazidime resistance. Samples were incubated with 100 μg/ml cefotaxime **(A)** or 20 μg/ml ceftazidime **(B)** for time periods of 0–12 h. **(C & D)** Optimization of cefotaxime and ceftazidime concentrations for detection of cefotaxime and ceftazidime resistance. Samples were incubated with 10–100 μg/ml of cefotaxime **(C)** or 5–50 μg/ml of ceftazidime **(D)** for 1 h. All experiments were repeated three-times in triplicate. Bars represent the mean proportion of cefotaxime or ceftazidime loss ± standard error of the mean. Incubation times do not include the additional preparation time (∼15 min). NS: Nonsignificant.

**Figure 2.**
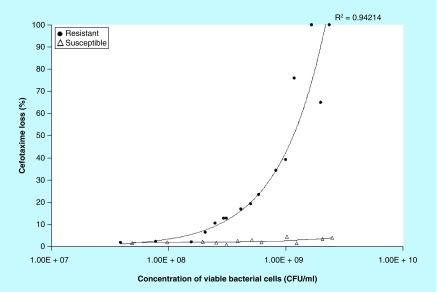
**Effect of bacterial concentration on cefotaxime loss.** Various concentrations of cefotaxime-resistant extended-spectrum β-lactamase- and AmpC-producing *E. coli* (circles) or cefotaxime-susceptible *E. coli* (triangles) were incubated with 20 μg/ml cefotaxime for 1 h and the loss of cefotaxime determined by the HPLC assay. Each data point is the mean of three technical replicates of assay; four different concentrations (tenfold dilutions) were assessed on four different occasions. The concentration of viable bacterial cells was determined for each experiment.

**Figure 3.**
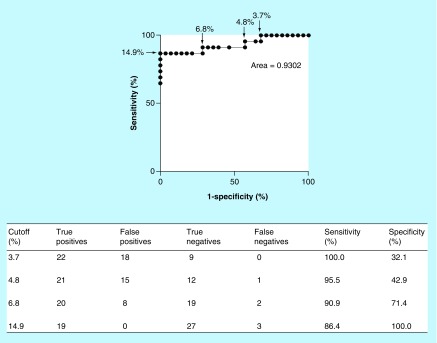
**Performance of the HPLC method for the detection of cefotaxime-resistant bacteria.** The receiver operating characteristic curve was calculated to show the sensitivity and specificity of the data from the proportion of cefotaxime loss in cefotaxime-susceptible (n = 27) and cefotaxime-resistant (n = 22) samples. The area under the curve was 0.9302.

Over the last decade there has been an alarming increase in the incidence of bloodstream infections caused by antibiotic-resistant organisms, especially *Enterobacteriaceae* and *Pseudomonas aeruginosa* [1]. In New Zealand, there is an epidemic of extended-spectrum β-lactamase (ESBL)-producing gram-negative bacteria that is rapidly depleting the therapeutic options [2]. The emergence of increased antimicrobial resistance is associated with an increased incidence of sepsis by an annual rate of 8–13% over the last decade [3,4]. Retrospective studies demonstrate that survival rates of patients with septic shock decrease by an average of 8% for every hour that effective antimicrobial therapy is delayed [5,6]. In patients with bacteremia who require Intensive Care Unit (ICU) admission and who are treated with appropriate antibiotics, attributable mortality is 28.4% [5]. Conversely, in patients treated with inappropriate antibiotics, mortality is 61.9% [5].

β-lactam antibiotics are frequently used for the empiric treatment of sepsis. Third-generation cephalosporins were developed to have a broad spectrum of activity against β-lactamase-producing gram-negative bacteria and are an important front-line therapy [7,8]. However, point mutations in the active site of widespread β-lactamases (TEM-1, TEM-2, SHV-1 and CTX-M) have enhanced their spectrum of catalytic activity to include the extended spectrum cephalosporins, penicillins and monobactams [8]. CTX-M-type β-lactamases have recently emerged as the most common type of ESBL and the most prominent cause of resistance to extended-spectrum cephalosporins [8]. Concern has also been raised about the increasing incidence of plasmid-mediated AmpC β-lactamases [9]. Importantly, bacteria encoding ESBLs and plasmid-mediated AmpC β-lactamases are often resistant to multiple classes of antibiotics as the β-lactamase genes are present in mobile genetic elements containing multiple other resistance genes [10].

To provide early and effective treatment and reduce the dissemination of resistant bacteria, rapid diagnostic tests are essential. Currently, susceptibility testing for cephalosporins and other antimicrobials relies upon phenotypic testing of isolates by standardized methods, such as broth microdilution and disk diffusion, and interpretation using clinical breakpoints [11]. These tests are time consuming and results may not be available for up to 24 h [12]. Therefore, there is an urgent need for robust diagnostic resistance tests that are faster than classical methods [12].

Here, we report an HPLC-based phenotypic assay to rapidly detect the breakdown of extended-spectrum cephalosporins by ESBL and AmpC β-lactamase-producing *Enterobacteriaceae*, allowing the detection of resistance within 90 min.

## Materials & methods

### Bacterial strains

*Escherichia coli* (*E. coli*) (American Type Culture Collection [ATCC]: 25922) was obtained from the New Zealand Culture Collection, The Institute of Environmental Science and Research. Clinical isolates were collected from blood cultures in the diagnostic laboratory; one isolate dispatched by the RCPA Quality Assurance Program was also included (*Yokenella regensburgei*). All isolates (n = 49) were identified by MALDI-ToF MS (Microflex LT, Bruker Daltonics). A mix of isolates resistant or susceptible to third-generation cephalopsorins were selected (Supplementary Table 1).

### HPLC method development

Chromatographic separation of cefotaxime (Hospira, Auckland, New Zealand) and ceftazidime (Alphapharm, Sydney, Australia) was performed on a C18 reverse phase column (150 × 4.60 mm, 5 μm) with a C18 guard column (4.0 × 3.0 mm) (both Phenomenex, CA, USA). The HPLC system (1260 Infinity, Agilent, CA, USA) was comprised of a quaternary pump system (G1311C), an auto sampler (G1329B), a column compartment (G1330B) and an UV/diode array detector (G1315D). The mobile phases, filtered and degassed, consisted of 0.1% formic acid (Sigma-Aldrich) in deionized water (18 MΩ resistivity, Milli-Q^®^ water purification system) and 0.1% formic acid in acetonitrile (Sigma-Aldrich). A linear gradient from 7.0 to 19.0% acetonitrile over 6 min and 19.0 to 40.0% from 6 to 13 min was used with a flow rate of 1.0 ml/min. Detection wavelength was 260 nm for both ceftazidime and cefotaxime at ambient temperature. Injection volume was 20 μl. Lab Chemstation (Version A.01.04, Agilent, CA, USA) was used for data acquisition and analysis. Quantification was based on the integration of the area underneath the peaks for cefotaxime (retention time 10.3 min) and ceftazidime (retention time 5.3 min). A standard curve was performed with each run.

### HPLC method validation

The method was validated according to the acceptance criteria of Industrial Guidance for Bioanalytical Analysis [13]. Standard solutions of cefotaxime (10–500 μg/ml) and ceftazidime (5–150 μg/ml) in deionized water were used to determine the standard curves (Table 1). To evaluate the inter- and intraday precision and accuracy of the HPLC method, three different concentrations (low, mid and high) of cefotaxime and ceftazidime were measured in six replicates over 3 days (Table 2). On each day freshly prepared standards were used to calculate the calibration curve. The intra and interday relative standard deviation of the assays was low, <3.2% for cefotaxime and <4.29% for ceftazidime and the bias was less than ±6.0%. The bacterial matrix, phosphate-buffered saline (PBS) was evaluated on three separate occasions with and without cefotaxime. The matrix did not have any affect on assay performance.

### Optimization of the HPLC assay for the detection of resistant bacteria

To determine whether cefotaxime and ceftazidime could be used to detect resistant bacteria, a clinical ESBL- and AmpC-producing strain of *E. coli* (clinical isolate R0) and a susceptible reference strain of *E. coli* (ATCC 25922) were resuspended in PBS, the turbidity adjusted to 4.0 McFarland (1.2 × 10^9^ CFUs/ml) and incubated with cefotaxime (10–100 μg/ml) or ceftazidime (5–50 μg/ml) for 0, 1, 2, 3 and 12 h at 37°C. The difference between the measured concentration and the starting concentration was calculated to determine the loss of cefotaxime or ceftazidime. The loss was reported as a proportion (%) of the starting concentration.

### Testing of isolates

Isolates were subcultured on Columbia blood agar (Fort Richard Laboratories, New Zealand) overnight. Isolated colonies were then resuspended in PBS and adjusted to 4.0 McFarland to which cefotaxime was added to make final concentration of 20 μg/ml. Suspensions were vortexed and incubated for 1 h at 37°C, then centrifuged, filtered and measured by HPLC assay.

### Phenotypic & genotypic characterization of clinical isolates

Disk diffusion testing was performed and interpreted by the Clinical and Laboratory Standards Institute (CLSI) method [11]. Mueller–Hinton agar (Fort Richard Laboratories, New Zealand) and cefotaxime, ceftazidime and cefepime-impregnated disks (MastDiscs™, Mast Diagnostics, UK) were used. Confirmatory ESBL-testing was performed by the CLSI method [11]. The AmpC and ESBL Detection Set (D68C, MastDiscs™, Mast Diagnostics, UK) was used to test for AmpC production in non-ESCHAPPM organisms.

Genotypic characterization of ESBL and plasmid-mediated AmpC enzymes was conducted using a published method [14]. Mastermixes of primers (IDT, IA, USA) were prepared for each multiplex or singleplex PCR reaction. PCR reactions were performed with KAPA2G Robust HotStart kit (Kapa Biosystems, MA, USA).

### Statistical analysis

Data were analyzed using Prism^®^ version 6.0 (GraphPad Software, CA, USA). Groups were compared with a two-way analysis of variance; with Tukey's multiple comparisons post-test. Receiver operating characteristic curve analysis was performed and sensitivities and specificities, relative to the CLSI disk diffusion method, calculated for each cutoff threshold of the HPLC assay.

## Results

Using 100 μg/ml of cefotaxime, there was an observable difference in the concentration of cefotaxime between samples with resistant bacteria and samples with susceptible bacteria after a 1-h incubation, (24.1% loss vs 6.5% loss), although this did not reach statistical significance (Figure 1A). Resistant samples were found to have a statistically greater cefotaxime loss after 2 h (47.4 vs 10.8%; p = 0.014) and 3 h (66.1 vs 17.3%; p = 0.001) compared with susceptible bacteria. Using 20 μg/ml of ceftazidime, there was no statistical difference in ceftazidime loss between resistant, susceptible and no bacteria samples after 1-, 2- and 3-h incubations (Figure 1B). However, at 12-h incubation, there was a statistically significant difference in the concentration of ceftazidime between samples with resistant bacteria and samples with susceptible bacteria (54.4 vs 6.2%; p = 0.001).

The effect of antibiotic concentration was then assessed to determine the concentration that would produce the greatest proportion of cefotaxime or ceftazidime loss with resistant *E. coli* after 1-h incubation (Figure 1C & D). Statistically significant differences between cefotaxime-resistant and cefotaxime-susceptible *E. coli* were present for all the tested concentrations of cefotaxime after 1-h incubation. At 10, 20 and 50 μg/ml resistant and susceptible *E. coli* were easily discriminated (10 μg/ml, 60.9 vs -18.2%; p < 0.0001; 20 μg/ml, 68.7 vs -5.4%; p < 0.0001; 50 μg/ml, 56.9 vs 2.1%; p = 0.0012); 20 μg/ml of cefotaxime provided the highest proportion of cefotaxime loss after 1-h incubation and had an acceptable level of accuracy in the no bacteria control. Thus, 20 μg/ml cefotaxime provided the greatest sensitivity for detecting resistant strains of bacteria after 1-h incubation. No statistical difference was observed between resistant and susceptible bacteria after 3-h incubation with varying concentrations of ceftazidime (Figure 1D). Hence, ceftazidime could not be used to accurately to differentiate between bacterial resistance mediated by ESBL and AmpC β-lactamase production, susceptible bacteria and no bacteria controls within the acceptable incubation period of 1–3 h.

Next, the bacterial concentration required to produce an observable effect on the cefotaxime concentration (20 μg/ml, 1 h) was assessed (Figure 2). Cefotaxime-resistant and cefotaxime-susceptible *E. coli* could be distinguished when the concentration of bacterial cells was between 2.5 × 10^8^ and 2.4 × 10^9^ CFU/ml. As the resistant *E. coli* concentration increased, the loss of cefotaxime increased until no cefotaxime peaks were detected (100% loss).

Isolates were phenotypically and genotypically characterized (Supplementary Tables 1 & 2, respectively). Overall, 45% (22/49) were cefotaxime resistant. CTX-M group 1 (11/22) and CTX-M group 9 (7/22) were the most common ESBLs. Plasmid-mediated AmpC β-lactamases were detected in 4/22 isolates; variants were from CIT families (CMY/LAT) (Supplementary Table 2).

### Evaluating the HPLC method for detecting cefotaxime-resistant bacteria

The characterized 49 bacterial strains were suspended in PBS and incubated with 20 μg/ml of cefotaxime for 1 h at 37°C and then prepared for HPLC analysis. A cutoff threshold of cefotaxime loss was determined using receiver operating characteristic curve data analysis to maximize the sensitivity and specificity of the assay (Figure 3). The optimal cutoff threshold for cefotaxime loss was ≥14.9% as this provided a high level of sensitivity at 86.4% and specificity at 100%. With the cutoff threshold of ≥14.9% the HPLC method correctly classified 19/22 of the gram-negative organisms that displayed phenotypic resistance to cefotaxime. This included 15/15 ESBL-mediated resistant strains and 3/3 ESBL- and AmpC-producing strains. The method failed to detect three cefotaxime-resistant strains, including two AmpC β-lactamase strains (R11 and R21) and one resistant *E. coli* strain that had no phenotypic evidence for an ESBL or AmpC (R18) (Supplementary Table 2). No susceptible bacteria were detected as cefotaxime resistant with this cutoff threshold.

## Conclusions & future perspective

We have developed an assay for the rapid detection of phenotypic resistance of Gram-negative bacteria to cefotaxime. This assay is based upon the detection by HPLC of the loss of cefotaxime in the presence of bacterial β-lactamases. This assay had a high sensitivity and specificity for the detection of resistance, especially that mediated by ESBL production. When combined with bacterial identification by MALDI-ToF MS, this assay could potentially enable the detection of cefotaxime-resistant bacteria directly from positive blood cultures within 90 min, almost a day early than direct susceptibility testing.

Rapid characterization of antibiotic resistance could facilitate improved patient outcomes while supporting antimicrobial stewardship [15]. In gram-negative pathogens, β-lactamase production remains the most important mechanism contributing to β-lactam resistance [16]. Third- and fourth- generation cephalosporins were developed as extended-spectrum antibiotics to overcome resistance mediated by common β-lactamases [17]. They are commonly used in empirical broad-spectrum antibiotic regimens to treat serious infections prior to culture and susceptibility results being available [18]. The guidelines for susceptibility testing set out by CLSI and European Committee on Antimicrobial Susceptibility Testing have recently removed the need to perform ESBL screening and confirmatory tests except for epidemiological or infection control purposes [19,20]. It is recommended that all the results from susceptibility testing be reported as tested, irrespective of whether the organism contains an ESBL or AmpC enzyme [20]. Therefore, it is important that any rapid phenotypic screen, such as the one outlined in this study, correlate with formal susceptibility testing results, rather than with the presence or the absence of particular resistance mechanisms.

ESBL and plasmid-mediated AmpC production are associated with multidrug resistance [21]. This is due to the accumulation of resistance genes on mobile genetic elements [21]. Therefore, detection of resistance to cefotaxime in organisms that are usually susceptible (e.g., *E. coli*, *K. pneumoniae*) is a useful early marker of multidrug resistance and allows early broadening of empiric therapy.

While hydrolysis of cefotaxime was found to be a useful early marker of ESBL production, ceftazidime was not. This likely reflects the mechanism of resistance in the organism used for method development, which contained a group 1 CTX-M and AmpC enzymes. CTX-M β-lactamases have an increased substrate affinity for cefotaxime compared with ceftazidime [22]. CTX-M-15 (group 1) and CTX-M-14 (group 9) are now the predominant ESBL subtypes in many parts of the world, including New Zealand [23]. This was reflected in our culture collection, where all ESBL-producing isolates were CTX-M group 1 (11/18) or group 9 (7/18). Of these all were phenotypically resistant to cefotaxime, while only 56% were resistant to ceftazidime and 72% to cefepime. Therefore, of those antibiotics tested cefotaxime is likely to be the best substrate for the rapid detection of ESBL production using the method described in this study. In areas where alternative ESBL enzymes (e.g., TEM, SHV, OXA) are more common, it may be prudent to test hydrolysis of both cefotaxime and ceftazidime [24,25].

Using cefotaxime, ESBL-mediated resistance was more readily detected than AmpC-mediated resistance, although few isolates with AmpC-mediated resistance were assessed (three in the absence of ESBL coproduction). This may reflect the relative affinity of the enzymes for cefotaxime [26]. Most AmpC-producing organisms are members of the ESCAPPM group, with the presence of the gene predicted by the bacterial identification [22], which can be rapidly obtained by MALDI-ToF MS [27,28], although phenotypic resistance requires constitutive AmpC expression. While plasmid-mediated AmpC production has been reported in *E. coli* and *K. pneumoniae*, it is less common than ESBL-production [29]. AmpC production in *E. coli* can also be due hyperproduction of the chromosomal gene due to a mutation of the promoter [22]; this may be the case in isolate R21 where no plasmid-associated AmpC gene was detected.

MALDI-ToF MS has also been used to detect β-lactamase-mediated resistance to β-lactam antibiotics [27,30–32]. Following a 1–2.5-h coincubation of gram-negative bacteria with a relevant antibiotic the ratio of hydrolyzed to nonhydrolyzed β-lactam could be used to classify resistance or susceptibility [27,33]. While MALDI-ToF MS is readily available in clinical microbiology laboratories, the mass of antibiotics and their hydrolysis products is below the normal detection range of the system and MALDI-ToF MS has limited capability to quantify molecules of low molecular mass (<1000 Da), which may reduce the sensitivity and accuracy of this approach [34,35]. In addition, small changes in instrument performance can impact on the detection of single peaks and subtle changes in mass, such as the detection of antibiotic hydrolysis products [36].

To improve sensitivity, Peaper *et al*. used LC-MS to monitor carbapenemase activity by quantifying the disappearance of ertapenem and the appearance of its hydrolyzed metabolite in a complex biological matrix [37]. Similarly, Grundt *et al*. developed an LC-MS assay to quantify the hydrolysis of ampicillin into penicilloic acid in the presence of β-lactamase-producing *E. coli* [34]. Cefotaxime has also been used as a marker in an LC-MS assay to quantify β-lactamase activities, achieving a sensitivity of 92.4% and specificity of 97.4% [38]. Compared with classic antibiotic susceptibility testing the assay significantly reduced the turnaround time but requires further evaluation with other *Enterobacteriaceae* [38].

The HPLC method described here provides an alternative to LC-MS, achieving rapid detection of resistance with high sensitivity. When cefotaxime loss ≥14.9% was used as the cutoff, cefotaxime resistance could be detected with 86.4% sensitivity and 100% specificity. This allowed the detection of 100% of ESBL-producing bacteria in approximately 1.5 h, thus saving 16–24 h compared with conventional phenotypic testing.

There are some limitations of the assay. First, a standard curve is required to quantify cefotaxime. Accurate quantification is important, as the absolute decrease in cefotaxime under the optimized conditions was small with some cefotaxime-resistant isolates. The amount of cefotaxime hydrolyzed, and hence the difference between susceptible and resistant isolates, could be enhanced by lengthening the incubation time or by increasing the bacterial concentration, although the latter is not possible if the assay is to be used directly on positive blood cultures. While assaying the hydrolysis product of cefotaxime would have been desirable, we were unable to detect it by HPLC; LC-MS would be required to measure hydrolysis products. Finally, all ESBL-producing *Enterobacteriaceae* included in the study contained a CTX-M-type enzyme. The performance of the assay may vary with other enzymes (e.g., TEM, SHV and OXA variants).

Before this assay can be applied in a clinical setting, it will need to be prospectively evaluated with *Enterobacteriaceae* from clinical blood cultures. A larger variety of ESBL- and AmpC-producing isolates should be assessed and the thresholds determined in this study validated. This was not performed due to the low incidence of bacteremia with ESBL- and AmpC-producing *Enterobacteriaceae* in our institution.

In conclusion, this is one of the first studies to use HPLC to screen for resistant bacteria. The method was able to detect cefotaxime resistance with high sensitivity within 90 min, 16–24 h earlier than conventional methods.

**Table 1.** **Summary of chromatographic parameters for detection of cefotaxime and ceftazidime^†^.**

**Analyte**	**λ (nm)**	**t_R_ mean value ± SD (min)**	**Calibration range (μg/ml)**	**Slope (95% CI)**	**Y-intercept (95% CI)**	**R^2^**	**LOD (ng/ml)**	**LOQ (ng/ml)**
Cefotaxime	260	10.71 ± 0.03	10–500	38.4 (36.9–39.9)	-194.2 (-549.0 to 160.8)	0.994	10.93	33.12
Ceftazidime	260	5.36 ± 0.03	5–150	44.1 (43.7–44.5)	29.36 (0.30 to 58.42)	>0.999	7.69	23.29

^†^Chromatographic information (detector wavelength [λ] and mean retention time [t_R_] ± [SD]), linearity details (calibration range, equation and R^2^), LOD and LOQ.

**Table 2.** **Intra and interday precision and inaccuracy for the quantification of cefotaxime and ceftazidime by HPLC.**

	**Intraday (n = 6)**			**Interday (n = 3)**		
**Theoretical concentration**	**Mean measured concentration ± SD**	**RSD (%)**	**Bias (%)**	**Mean measured concentration ± SD**	**RSD (%)**	**Bias (%)**
***Cefotaxime (μg/ml)***						
30	29.06 ± 0.82	2.83	3.13	29.44 ± 0.94	3.20	1.85
100	99.7 ± 2.3	2.34	0.29	100.8 ± 2.8	2.76	-0.82
400	399.4 ± 10.7	2.68	0.15	398.9 ± 8.5	2.12	0.27
***Ceftazidime (μg/ml)***						
15	15.14 ± 0.21	1.39	-0.91	15.25 ± 0.57	3.75	-1.65
80	80.1 ± 3.4	4.29	-0.07	81.3 ± 2.3	2.8	-1.63
120	122.57 ± 0.67	0.55	-2.14	121.3 ± 1.7	1.39	-1.06

**Executive summary**

The emergence of bacterial resistance to antimicrobials is a significant global health challenge.
Rapid identification of resistant strains will support antimicrobial stewardship and improve treatment efficacy.
Hydrolysis of cefotaxime was found to be an effective marker of cefotaxime resistance in *Enterbacteriaceae*, especially when resistance was mediated by an extended-spectrum β-lactamases.
Using HPLC, resistance to cefotaxime could be detected with high sensitivity within 90 min.
