# Supplementary material for: The rapid detection of cefotaxime-resistant Enterobacteriaceae by HPLC
Source: Future Sci OA. 2016 Sep 9;2(4):FSO142. doi: 10.4155/fsoa-2016-0042 (PMC5242201; doi:10.4155/fsoa-2016-0042)
Supplement: Supplementary file 1 [file fsoa-02-143-s1.docx]

**Supplementary Table 1. Phenotypic characterisation of the isolates**

| Lab code | Species identification | Resistance classification (CLSI) | Phenotypic resistance or susceptibility to: | | | Cefotaxime loss (%) |
| --- | --- | --- | --- | --- | --- | --- |
|  |  |  | Cefotaxime (CTX 30) | Ceftazidime (CAZ 30) | Cefepime (CPM 30) |  |
| S control | *E. coli*  (ATCC 25922) | Susceptible | Susceptible | Susceptible | Susceptible | 5.0 |
| R0 | *E. coli* | ESBL and AmpC^a^ | Resistant | Intermediate | Resistant | 100 |
| R1 | *E. coli* | ESBL | Resistant | Resistant | Resistant | 100 |
| R2 | *E. coli* | ESBL | Resistant | Susceptible | Susceptible | 41.9 |
| R3 | *E. coli* | ESBL and AmpC^a^ | Resistant | Resistant | Resistant | 100 |
| R4 | *E. coli* | ESBL | Resistant | Resistant | Resistant | 100 |
| R5 | *E. coli* | ESBL | Resistant | Susceptible | Susceptible | 100 |
| R6 | *E. coli* | ESBL and AmpC^a^ | Resistant | Resistant | Resistant | 100 |
| R7 | *E. coli* | ESBL | Resistant | Intermediate | Susceptible | 43.7 |
| R8 | *E. coli* | AmpC^a^ | Resistant | Resistant | Susceptible | 100 |
| R9 | *E. coli* | ESBL | Resistant | Intermediate | Susceptible | 100 |
| R10 | *K. pneumoniae* | ESBL | Resistant | Resistant | Resistant | 75.5 |
| R11 | *E. cloacae* | AmpC^b^ | Resistant | Resistant | Susceptible | 3.8 |
| R12 | *K. pneumoniae* | ESBL | Resistant | Intermediate | Resistant | 100 |
| R13 | *C. braakii* | ESBL | Resistant | Resistant | Resistant | 15.5 |
| R14 | *E. coli* | ESBL | Resistant | Resistant | Resistant | 100 |
| R15 | *E. coli* | ESBL | Resistant | Susceptible | Susceptible | 100 |
| R16 | *E. cloacae* | ESBL | Resistant | Resistant | Resistant | 91.6 |
| R17 | *K. pneumoniae* | ESBL | Resistant | Resistant | Resistant | 100 |
| R18 | *E. coli* | Not classified^c^ | Resistant | Intermediate | Susceptible | 7.1 |
| R19 | *E. coli* | ESBL | Resistant | Resistant | Resistant | 95.1 |
| R20 | *E. coli* | ESBL | Resistant | Intermediate | Resistant | 100 |
| R21 | *E. coli* | AmpC^a^ | Resistant | Resistant | Susceptible | 4.9 |
| S1 | *E. coli* | Susceptible | Susceptible | Susceptible | Susceptible | 1.1 |
| S2 | *E. coli* | Susceptible | Susceptible | Susceptible | Susceptible | 1.2 |
| S3 | *E. coli* | Susceptible | Susceptible | Susceptible | Susceptible | 2.6 |
| S4 | *E. coli* | Susceptible | Susceptible | Susceptible | Susceptible | 4.7 |
| S5 | *E. coli* | Susceptible | Susceptible | Susceptible | Susceptible | -1.6 |
| S6 | *C. koseri* | Susceptible | Susceptible | Susceptible | Susceptible | 1.6 |
| S7 | *E. coli* | Susceptible | Susceptible | Susceptible | Susceptible | 14.2 |
| S8 | *E. coli* | Susceptible | Susceptible | Susceptible | Susceptible | 5.0 |
| S9 | *E. coli* | Susceptible | Susceptible | Susceptible | Susceptible | 8.2 |
| S10 | *E. coli* | Susceptible | Susceptible | Susceptible | Susceptible | 3.8 |
| S11 | *E. coli* | Susceptible | Susceptible | Susceptible | Susceptible | 5.3 |
| S12 | *E. coli* | Susceptible | Susceptible | Susceptible | Susceptible | 5.0 |
| S13 | *E. coli* | Susceptible | Susceptible | Susceptible | Susceptible | 10.0 |
| S14 | *E. coli* | Susceptible | Susceptible | Susceptible | Susceptible | 7.5 |
| S15 | *E. coli* | Susceptible | Susceptible | Susceptible | Susceptible | 8.5 |
| S16 | *P. mirabilis* | Susceptible | Susceptible | Susceptible | Susceptible | 5.3 |
| S17 | *E. coli* | Susceptible | Susceptible | Susceptible | Susceptible | 8.3 |
| S18 | *E. coli* | Susceptible | Susceptible | Susceptible | Susceptible | 7.5 |
| S19 | *P. mirabilis* | Susceptible | Susceptible | Susceptible | Susceptible | 0.2 |
| S20 | *E. coli* | Susceptible | Susceptible | Susceptible | Susceptible | 6.1 |
| S21 | *P. mirabilis* | Susceptible | Susceptible | Susceptible | Susceptible | 2.6 |
| S22 | *K. pneumoniae* | Susceptible | Susceptible | Susceptible | Susceptible | 3.5 |
| S23 | *E. coli* | Susceptible | Susceptible | Susceptible | Susceptible | 6.9 |
| S24 | *E. coli* | Susceptible | Susceptible | Susceptible | Susceptible | 5.7 |
| S25 | *Y. regensburgei* | AmpC ^b^ | Susceptible | Susceptible | Susceptible | 2.1 |
| S26 | *M. morganii* | AmpC^b^ | Susceptible | Susceptible | Susceptible | 4.5 |
|  |  |  |  |  |  |  |

^a^ AmpC resistance was confirmed by MAST^TM^ discs. ^b^ Chromosomal AmpC not tested with MAST^TM^ discs. ^c^ Negative ESBL confirmatory test with cefotaxime/ceftazidime/cefepime + clavulanic acid and no AmpC present as determined by MAST^TM^ discs. **Supplementary Table 2. Genotypic characterisation of the isolates**

| Lab code | Species identification | Phenotypic resistance classification (CLSI) | Mulitplex I: TEM, SHV and OXA-1-like | Mulitplex II: CTX-M group 1, group 2 and group 9 | | Singleplex I: CTX-M group 8/25 | Multiplex III: ACC, FOX, MOX, CIT and EBC |
| --- | --- | --- | --- | --- | --- | --- | --- |
| S control | *E. coli*  (ATCC 25922) | Susceptible | Negative | | Negative | Negative | Negative |
| R0 | *E. coli* | ESBL and AmpC | TEM & OXA-1-like | | CTX-M Group 1 | Negative | CMY/LAT |
| R1 | *E. coli* | ESBL | TEM | | CTX-M Group 1 | Negative | Negative |
| R2 | *E. coli* | ESBL | TEM | | CTX-M Group 9 | Negative | Negative |
| R3 | *E. coli* | ESBL and AmpC | TEM | | CTX-M Group 1 | Negative | CMY/LAT |
| R4 | *E. coli* | ESBL | TEM & OXA-1-like | | CTX-M Group 1 | Negative | Negative |
| R5 | *E. coli* | ESBL | TEM | | CTX-M Group 9 | Negative | Negative |
| R6 | *E. coli* | ESBL and AmpC | TEM | | CTX-M Group 1 | Negative | CMY/LAT |
| R7 | *E. coli* | ESBL | TEM | | CTX-M Group 9 | Negative | Negative |
| R8 | *E. coli* | AmpC | TEM | | Negative | Negative | CMY/LAT |
| R9 | *E. coli* | ESBL | TEM | | CTX-M Group 9 | Negative | Negative |
| R10 | *K. pneumoniae* | ESBL | TEM & OXA-1-like | | CTX-M Group 1 | Negative | Negative |
| R11 | *E. cloacae* | AmpC | TEM | | Negative | Negative | Negative |
| R12 | *K. pneumoniae* | ESBL | TEM | | CTX-M Group 1 | Negative | Negative |
| R13 | *C. braakii* | ESBL | TEM | | CTX-M Group 9 | Negative | Negative |
| R14 | *E. coli* | ESBL | TEM | | CTX-M Group 1 | Negative | Negative |
| R15 | *E. coli* | ESBL | TEM | | CTX-M Group 9 | Negative | Negative |
| R16 | *E. cloacae* | ESBL | TEM & OXA-1-like | | CTX-M Group 1 | Negative | Negative |
| R17 | *K. pneumoniae* | ESBL | TEM & OXA-1-like | | CTX-M Group 1 | Negative | Negative |
| R18 | *E. coli* | Not classified | TEM | | Negative | Negative | Negative |
| R19 | *E. coli* | ESBL | TEM & OXA-1-like | | CTX-M Group 1 | Negative | Negative |
| R20 | *E. coli* | ESBL | TEM | | CTX-M Group 9 | Negative | Negative |
| R21 | *E. coli* | AmpC | TEM | | Negative | Negative | Negative |
